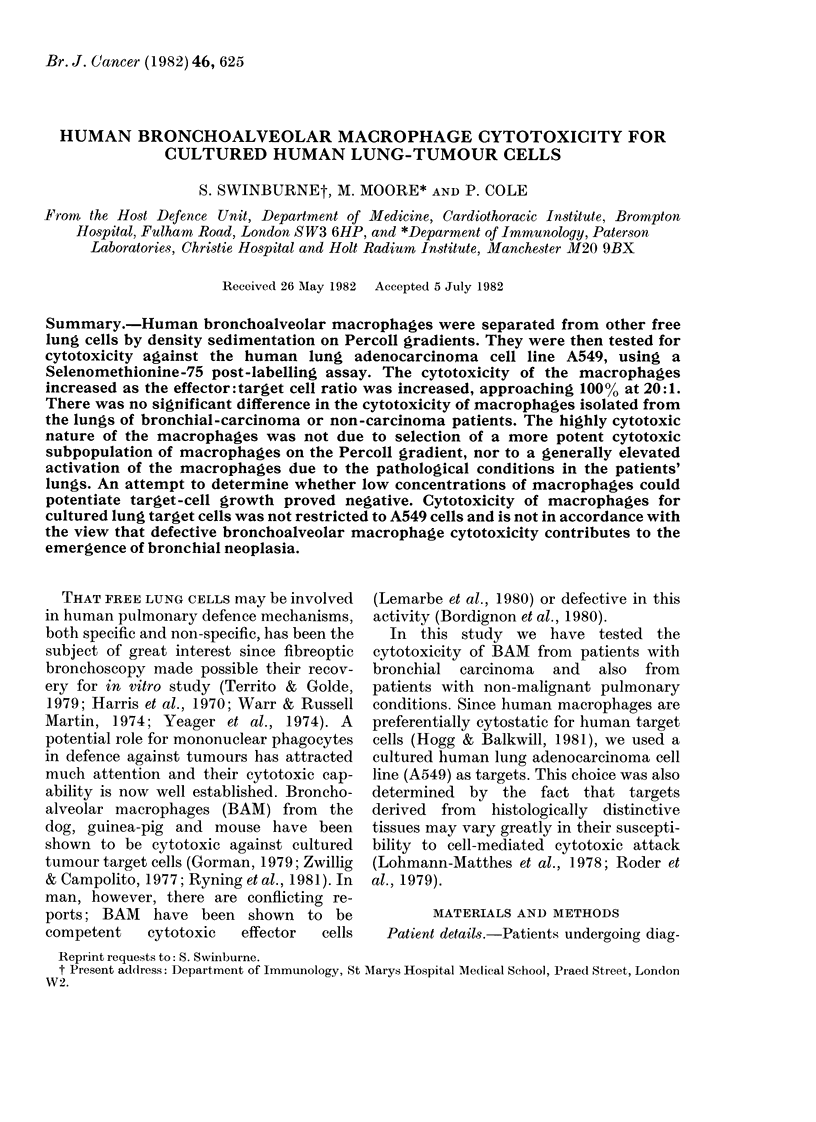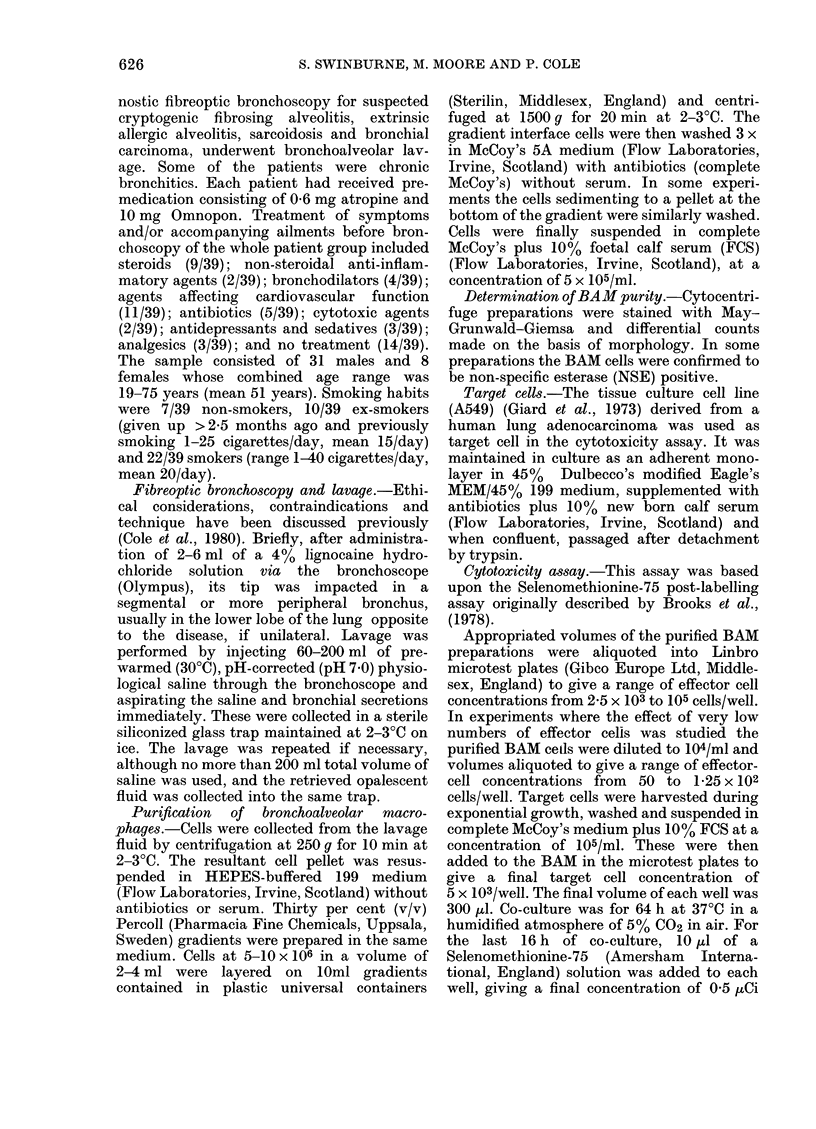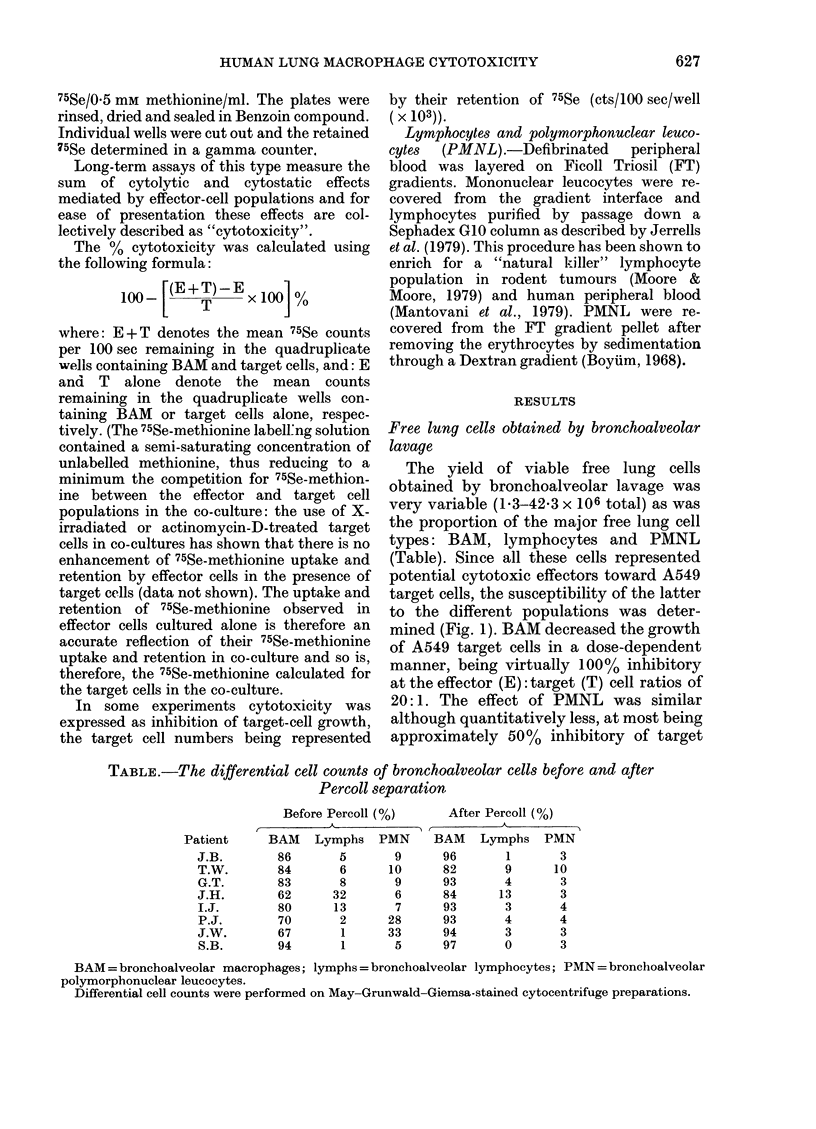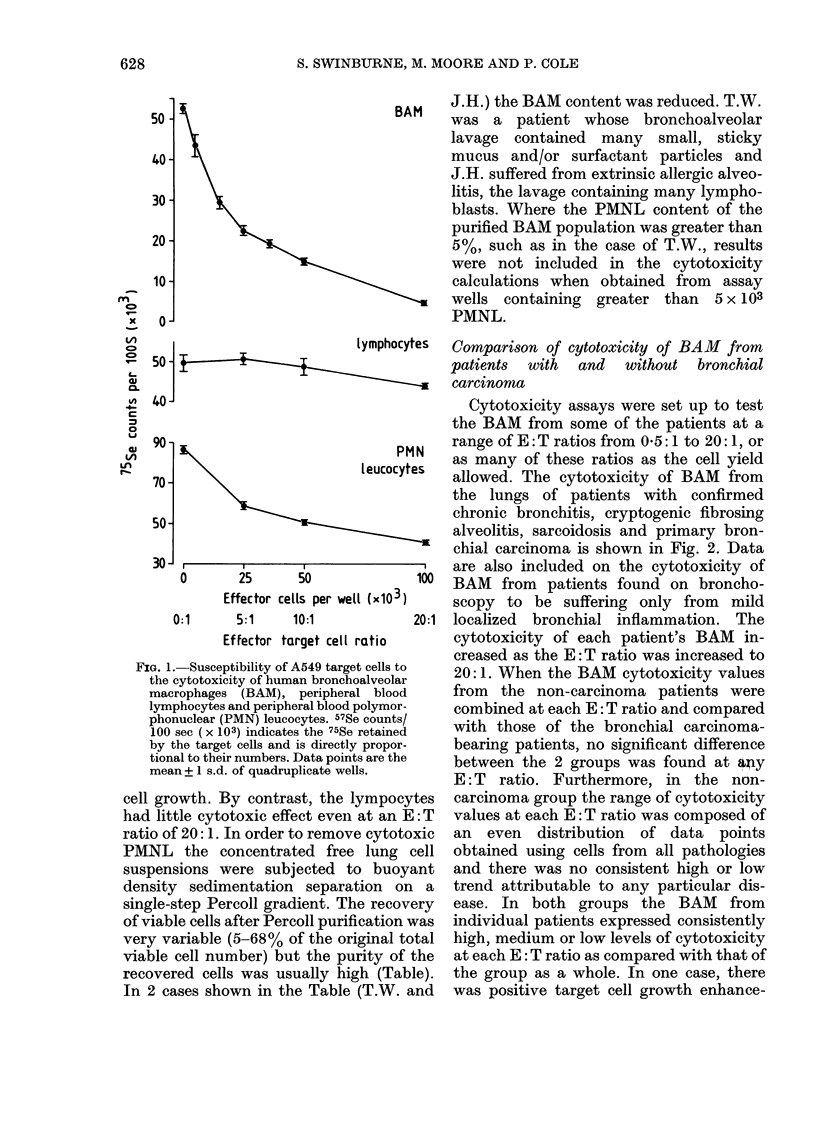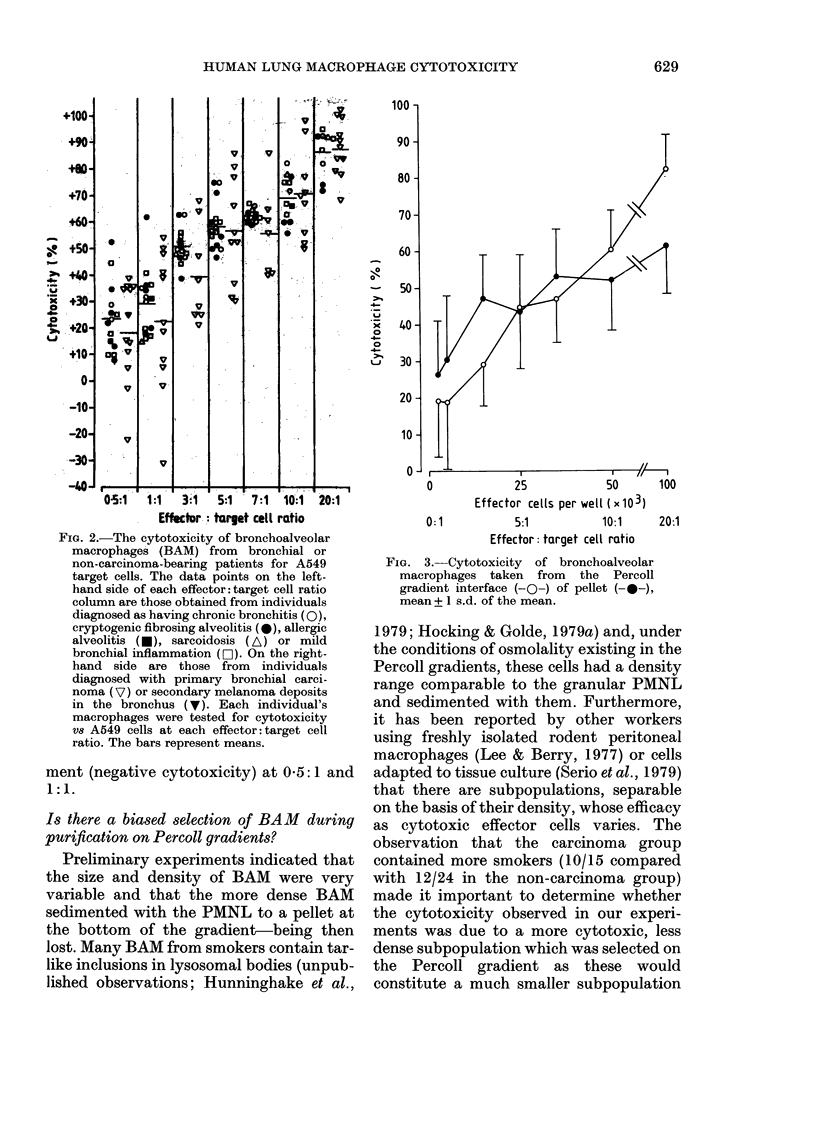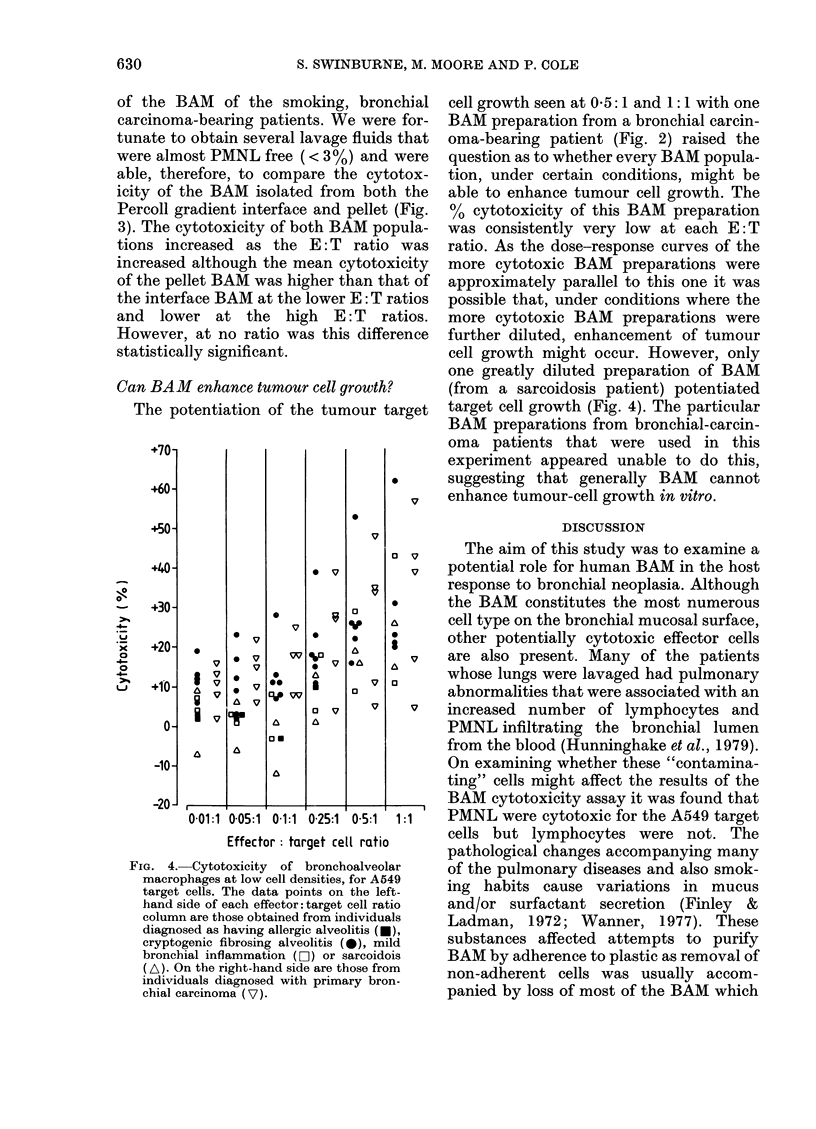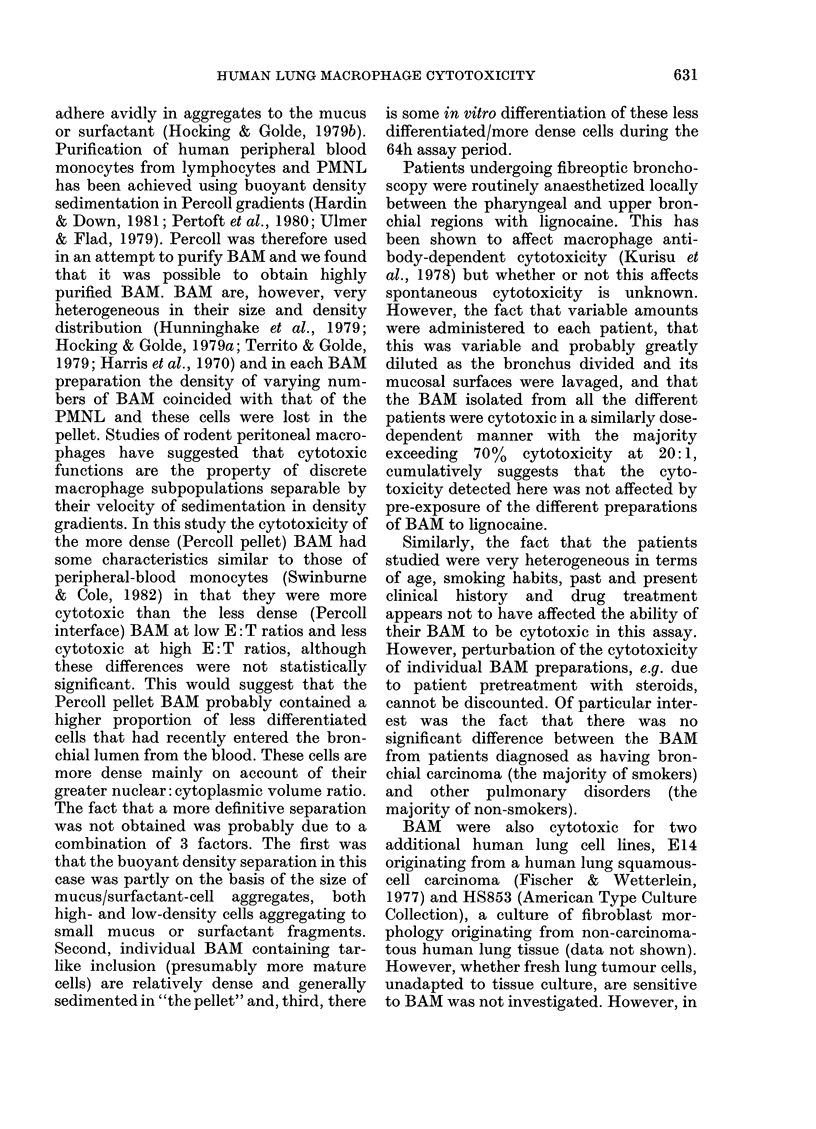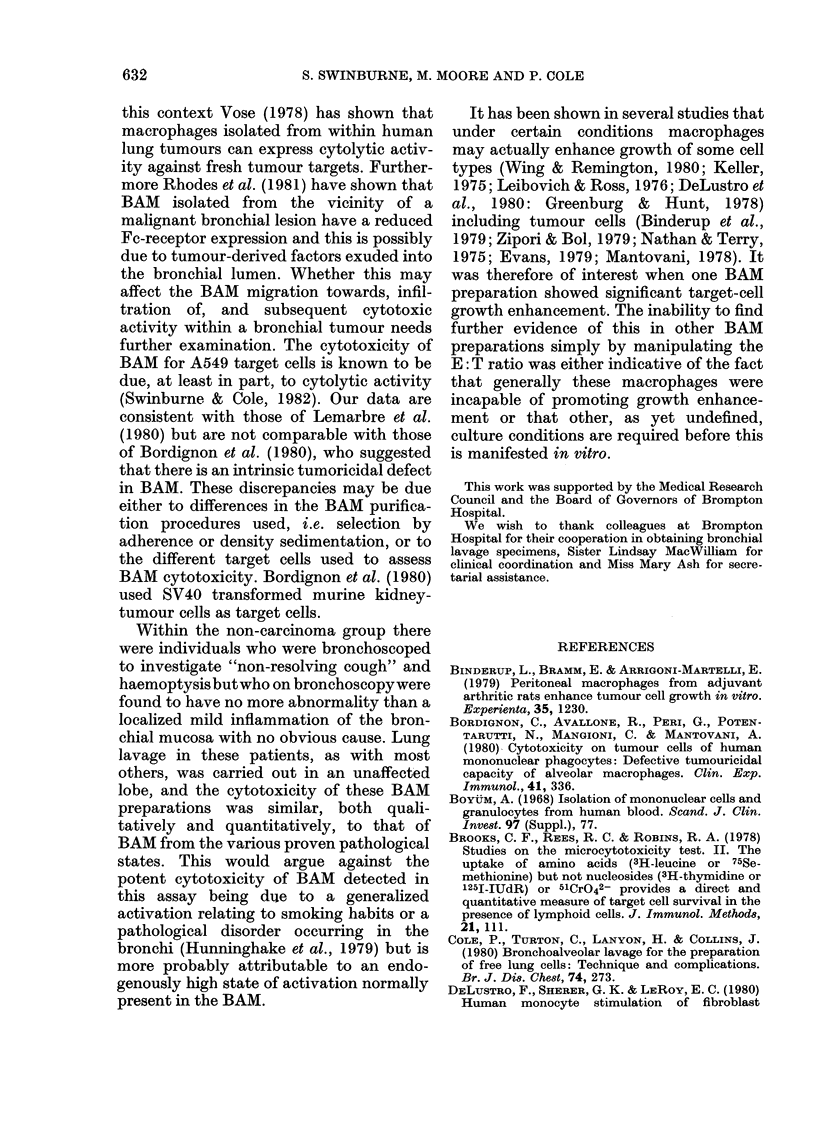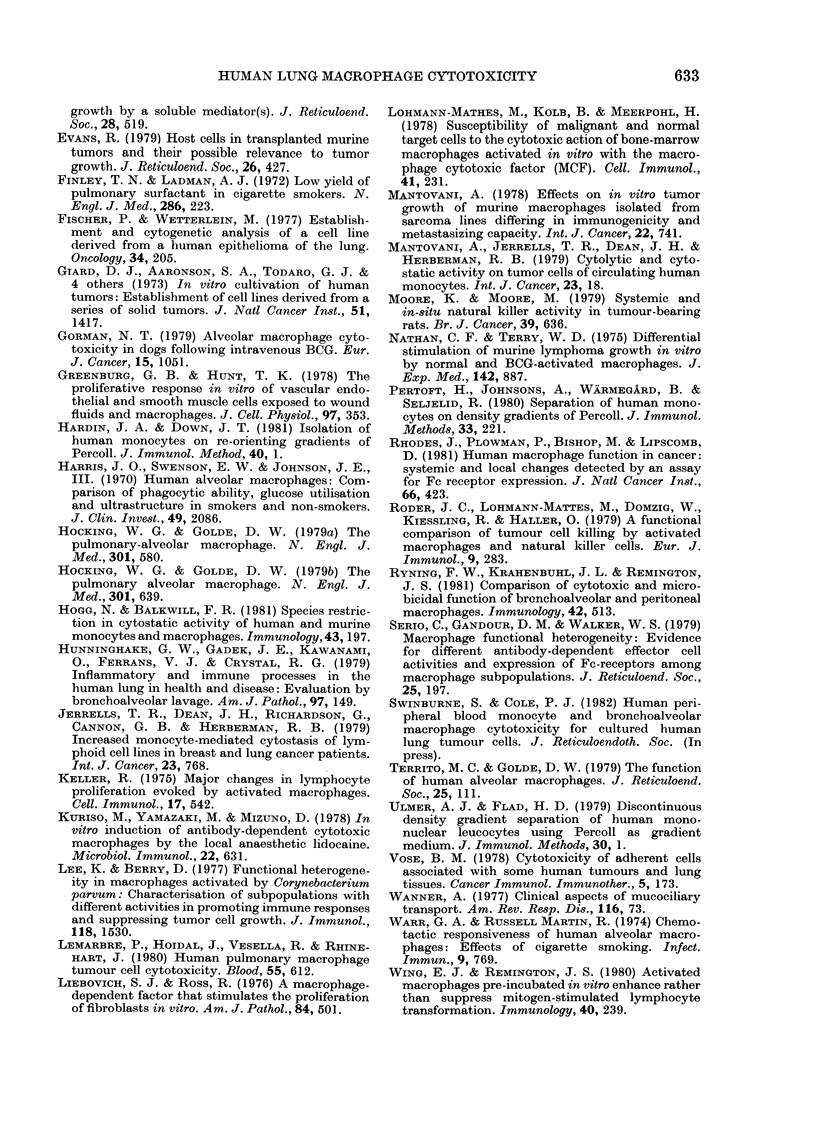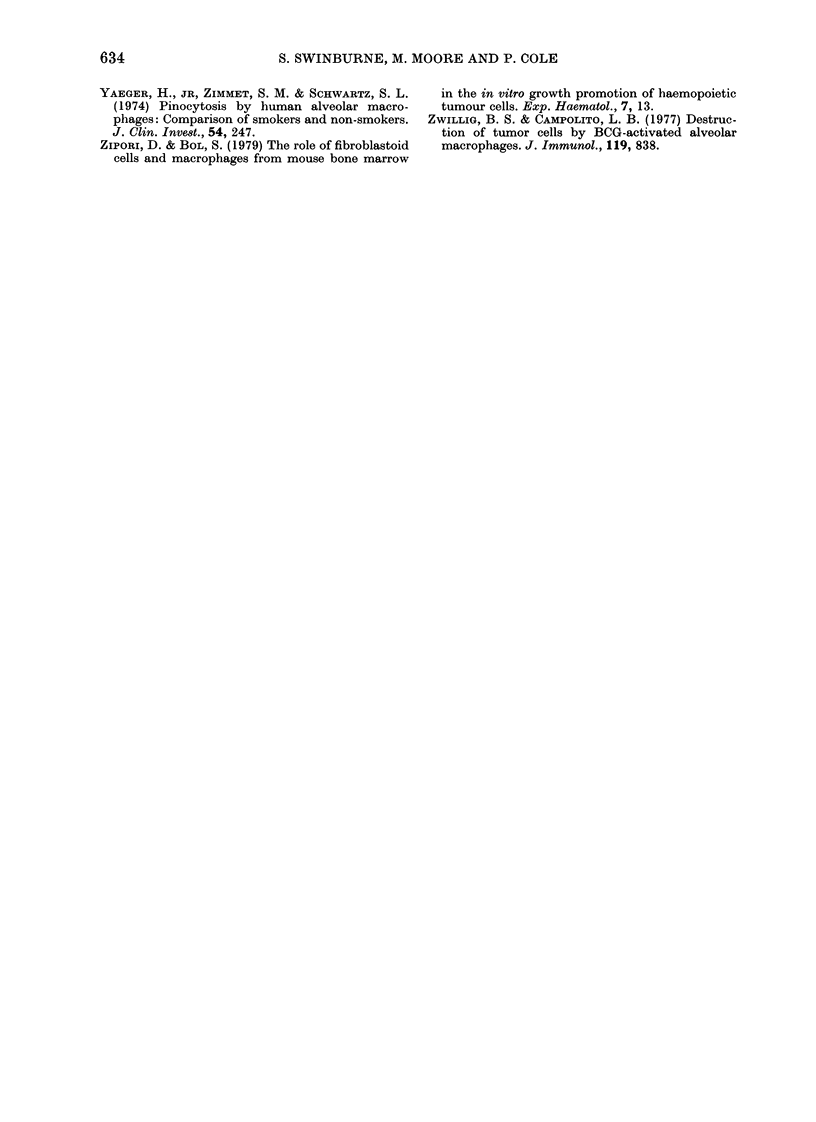# Human bronchoalveolar macrophage cytotoxicity for cultured human lung-tumour cells.

**DOI:** 10.1038/bjc.1982.247

**Published:** 1982-10

**Authors:** S. Swinburne, M. Moore, P. Cole

## Abstract

Human bronchoalveolar macrophages were separated from other free lung cells by density sedimentation on Percoll gradients. They were then tested for cytotoxicity against the human lung adenocarcinoma cell line A549, using a Selenomethionine-75 post-labelling assay. The cytotoxicity of the macrophages increased as the effector:target cell ratio was increased, approaching 100% at 20:1. There was no significant difference in the cytotoxicity of macrophages isolated from the lungs of bronchial-carcinoma or non-carcinoma patients. The highly cytotoxic nature of the macrophages was not due to selection of a more potent cytotoxic subpopulation of macrophages on the Percoll gradient, nor to a generally elevated activation of the macrophages due to the pathological conditions in the patients' lungs. An attempt to determine whether low concentrations of macrophages could potentiate target-cell growth proved negative. Cytotoxicity of macrophages for cultured lung target cells was not restricted to A549 cells and is not in accordance with the view that defective bronchoalveolar macrophage cytotoxicity contributes to the emergence of bronchial neoplasia.


*Br. J. Cancer* (1982) **46**, 625

### HUMAN BRONCHOALVEOLAR MACROPHAGE CYTOTOXICITY FOR CULTURED HUMAN LUNG-TUMOUR CELLS

S. SWINBURNE†, M. MOORE* AND P. COLE

*From the Host Defence Unit, Department of Medicine, Cardiothoracic Institute, Brompton Hospital, Fulham Road, London SW3 6HP, and *Deparment of Immunology, Paterson Laboratories, Christie Hospital and Holt Radium Institute, Manchester M20 9BX*

Received 26 May 1982 Accepted 5 July 1982

**Summary.—Human bronchoalveolar macrophages were separated from other free lung cells by density sedimentation on Percoll gradients. They were then tested for cytotoxicity against the human lung adenocarcinoma cell line A549, using a Selenomethionine-75 post-labelling assay. The cytotoxicity of the macrophages increased as the effector:target cell ratio was increased, approaching 100% at 20:1. There was no significant difference in the cytotoxicity of macrophages isolated from the lungs of bronchial-carcinoma or non-carcinoma patients. The highly cytotoxic nature of the macrophages was not due to selection of a more potent cytotoxic subpopulation of macrophages on the Percoll gradient, nor to a generally elevated activation of the macrophages due to the pathological conditions in the patients' lungs. An attempt to determine whether low concentrations of macrophages could potentiate target-cell growth proved negative. Cytotoxicity of macrophages for cultured lung target cells was not restricted to A549 cells and is not in accordance with the view that defective bronchoalveolar macrophage cytotoxicity contributes to the emergence of bronchial neoplasia.**

THAT FREE LUNG CELLS may be involved in human pulmonary defence mechanisms, both specific and non-specific, has been the subject of great interest since fibreoptic bronchoscopy made possible their recovery for *in vitro* study (Territo & Golde, 1979; Harris *et al.*, 1970; Warr & Russell Martin, 1974; Yeager *et al.*, 1974). A potential role for mononuclear phagocytes in defence against tumours has attracted much attention and their cytotoxic capability is now well established. Bronchoalveolar macrophages (BAM) from the dog, guinea-pig and mouse have been shown to be cytotoxic against cultured tumour target cells (Gorman, 1979; Zwillig & Campolito, 1977; Ryning *et al.*, 1981). In man, however, there are conflicting reports; BAM have been shown to be competent cytotoxic effector cells (Lemarbe *et al.*, 1980) or defective in this activity (Bordignon *et al.*, 1980).

In this study we have tested the cytotoxicity of BAM from patients with bronchial carcinoma and also from patients with non-malignant pulmonary conditions. Since human macrophages are preferentially cytostatic for human target cells (Hogg & Balkwill, 1981), we used a cultured human lung adenocarcinoma cell line (A549) as targets. This choice was also determined by the fact that targets derived from histologically distinctive tissues may vary greatly in their susceptibility to cell-mediated cytotoxic attack (Lohmann-Matthes *et al.*, 1978; Roder *et al.*, 1979).

### MATERIALS AND METHODS

*Patient details.*—Patients undergoing diag-

Reprint requests to: S. Swinburne.

† Present address: Department of Immunology, St Marys Hospital Medical School, Praed Street, London W2.

nostic fibreoptic bronchoscopy for suspected cryptogenic fibrosing alveolitis, extrinsic allergic alveolitis, sarcoidosis and bronchial carcinoma, underwent bronchoalveolar lavage. Some of the patients were chronic bronchitics. Each patient had received premedication consisting of 0·6 mg atropine and 10 mg Omnopon. Treatment of symptoms and/or accompanying ailments before bronchoscopy of the whole patient group included steroids (9/39); non-steroidal anti-inflammatory agents (2/39); bronchodilators (4/39); agents affecting cardiovascular function (11/39); antibiotics (5/39); cytotoxic agents (2/39); antidepressants and sedatives (3/39); analgesics (3/39); and no treatment (14/39). The sample consisted of 31 males and 8 females whose combined age range was 19–75 years (mean 51 years). Smoking habits were 7/39 non-smokers, 10/39 ex-smokers (given up $>2{\cdot}5$ months ago and previously smoking 1–25 cigarettes/day, mean 15/day) and 22/39 smokers (range 1–40 cigarettes/day, mean 20/day).

*Fibreoptic bronchoscopy and lavage.*—Ethical considerations, contraindications and technique have been discussed previously (Cole *et al.*, 1980). Briefly, after administration of 2–6 ml of a 4% lignocaine hydrochloride solution *via* the bronchoscope (Olympus), its tip was impacted in a segmental or more peripheral bronchus, usually in the lower lobe of the lung opposite to the disease, if unilateral. Lavage was performed by injecting 60–200 ml of prewarmed (30°C), pH-corrected (pH 7·0) physiological saline through the bronchoscope and aspirating the saline and bronchial secretions immediately. These were collected in a sterile siliconized glass trap maintained at 2–3°C on ice. The lavage was repeated if necessary, although no more than 200 ml total volume of saline was used, and the retrieved opalescent fluid was collected into the same trap.

*Purification of bronchoalveolar macrophages.*—Cells were collected from the lavage fluid by centrifugation at 250 *g* for 10 min at 2–3°C. The resultant cell pellet was resuspended in HEPES-buffered 199 medium (Flow Laboratories, Irvine, Scotland) without antibiotics or serum. Thirty per cent (v/v) Percoll (Pharmacia Fine Chemicals, Uppsala, Sweden) gradients were prepared in the same medium. Cells at $5\text{–}10 \times 10^6$ in a volume of 2–4 ml were layered on 10ml gradients contained in plastic universal containers (Sterilin, Middlesex, England) and centrifuged at 1500 *g* for 20 min at 2–3°C. The gradient interface cells were then washed 3 × in McCoy's 5A medium (Flow Laboratories, Irvine, Scotland) with antibiotics (complete McCoy's) without serum. In some experiments the cells sedimenting to a pellet at the bottom of the gradient were similarly washed. Cells were finally suspended in complete McCoy's plus 10% foetal calf serum (FCS) (Flow Laboratories, Irvine, Scotland), at a concentration of $5 \times 10^5$/ml.

*Determination of BAM purity.*—Cytocentrifuge preparations were stained with May–Grunwald–Giemsa and differential counts made on the basis of morphology. In some preparations the BAM cells were confirmed to be non-specific esterase (NSE) positive.

*Target cells.*—The tissue culture cell line (A549) (Giard *et al.*, 1973) derived from a human lung adenocarcinoma was used as target cell in the cytotoxicity assay. It was maintained in culture as an adherent monolayer in 45% Dulbecco's modified Eagle's MEM/45% 199 medium, supplemented with antibiotics plus 10% new born calf serum (Flow Laboratories, Irvine, Scotland) and when confluent, passaged after detachment by trypsin.

*Cytotoxicity assay.*—This assay was based upon the Selenomethionine-75 post-labelling assay originally described by Brooks *et al.*, (1978).

Appropriated volumes of the purified BAM preparations were aliquoted into Linbro microtest plates (Gibco Europe Ltd, Middlesex, England) to give a range of effector cell concentrations from $2{\cdot}5 \times 10^3$ to $10^5$ cells/well. In experiments where the effect of very low numbers of effector cells was studied the purified BAM cells were diluted to $10^4$/ml and volumes aliquoted to give a range of effector-cell concentrations from 50 to $1{\cdot}25 \times 10^2$ cells/well. Target cells were harvested during exponential growth, washed and suspended in complete McCoy's medium plus 10% FCS at a concentration of $10^5$/ml. These were then added to the BAM in the microtest plates to give a final target cell concentration of $5 \times 10^3$/well. The final volume of each well was 300 $\mu$l. Co-culture was for 64 h at 37°C in a humidified atmosphere of 5% $CO_2$ in air. For the last 16 h of co-culture, 10 $\mu$l of a Selenomethionine-75 (Amersham International, England) solution was added to each well, giving a final concentration of 0·5 $\mu$Ci

$^{75}$Se/0·5 mM methionine/ml. The plates were rinsed, dried and sealed in Benzoin compound. Individual wells were cut out and the retained $^{75}$Se determined in a gamma counter.

Long-term assays of this type measure the sum of cytolytic and cytostatic effects mediated by effector-cell populations and for ease of presentation these effects are collectively described as "cytotoxicity".

The % cytotoxicity was calculated using the following formula:

$$100 - \left[\frac{(E+T)-E}{T} \times 100\right] \%$$

where: E+T denotes the mean $^{75}$Se counts per 100 sec remaining in the quadruplicate wells containing BAM and target cells, and: E and T alone denote the mean counts remaining in the quadruplicate wells containing BAM or target cells alone, respectively. (The $^{75}$Se-methionine labelling solution contained a semi-saturating concentration of unlabelled methionine, thus reducing to a minimum the competition for $^{75}$Se-methionine between the effector and target cell populations in the co-culture: the use of X-irradiated or actinomycin-D-treated target cells in co-cultures has shown that there is no enhancement of $^{75}$Se-methionine uptake and retention by effector cells in the presence of target cells (data not shown). The uptake and retention of $^{75}$Se-methionine observed in effector cells cultured alone is therefore an accurate reflection of their $^{75}$Se-methionine uptake and retention in co-culture and so is, therefore, the $^{75}$Se-methionine calculated for the target cells in the co-culture.

In some experiments cytotoxicity was expressed as inhibition of target-cell growth, the target cell numbers being represented by their retention of $^{75}$Se (cts/100 sec/well ($\times 10^3$)).

*Lymphocytes and polymorphonuclear leucocytes (PMNL).*—Defibrinated peripheral blood was layered on Ficoll Triosil (FT) gradients. Mononuclear leucocytes were recovered from the gradient interface and lymphocytes purified by passage down a Sephadex G10 column as described by Jerrells *et al.* (1979). This procedure has been shown to enrich for a "natural killer" lymphocyte population in rodent tumours (Moore & Moore, 1979) and human peripheral blood (Mantovani *et al.*, 1979). PMNL were recovered from the FT gradient pellet after removing the erythrocytes by sedimentation through a Dextran gradient (Boyüm, 1968).

### RESULTS

### *Free lung cells obtained by bronchoalveolar lavage*

The yield of viable free lung cells obtained by bronchoalveolar lavage was very variable (1·3–42·3 $\times 10^6$ total) as was the proportion of the major free lung cell types: BAM, lymphocytes and PMNL (Table). Since all these cells represented potential cytotoxic effectors toward A549 target cells, the susceptibility of the latter to the different populations was determined (Fig. 1). BAM decreased the growth of A549 target cells in a dose-dependent manner, being virtually 100% inhibitory at the effector (E):target (T) cell ratios of 20:1. The effect of PMNL was similar although quantitatively less, at most being approximately 50% inhibitory of target

TABLE.—*The differential cell counts of bronchoalveolar cells before and after Percoll separation*

| | Before Percoll (%) | | | After Percoll (%) | | |
|---|---|---|---|---|---|---|
| Patient | BAM | Lymphs | PMN | BAM | Lymphs | PMN |
| J.B. | 86 | 5 | 9 | 96 | 1 | 3 |
| T.W. | 84 | 6 | 10 | 82 | 9 | 10 |
| G.T. | 83 | 8 | 9 | 93 | 4 | 3 |
| J.H. | 62 | 32 | 6 | 84 | 13 | 3 |
| I.J. | 80 | 13 | 7 | 93 | 3 | 4 |
| P.J. | 70 | 2 | 28 | 93 | 4 | 4 |
| J.W. | 67 | 1 | 33 | 94 | 3 | 3 |
| S.B. | 94 | 1 | 5 | 97 | 0 | 3 |

BAM = bronchoalveolar macrophages; lymphs = bronchoalveolar lymphocytes; PMN = bronchoalveolar polymorphonuclear leucocytes.

Differential cell counts were performed on May–Grunwald–Giemsa-stained cytocentrifuge preparations.

FIG. 1.—Susceptibility of A549 target cells to the cytotoxicity of human bronchoalveolar macrophages (BAM), peripheral blood lymphocytes and peripheral blood polymorphonuclear (PMN) leucocytes. $^{57}$Se counts/100 sec ( $\times 10^3$) indicates the $^{75}$Se retained by the target cells and is directly proportional to their numbers. Data points are the mean $\pm$ 1 s.d. of quadruplicate wells.

cell growth. By contrast, the lympocytes had little cytotoxic effect even at an E:T ratio of 20:1. In order to remove cytotoxic PMNL the concentrated free lung cell suspensions were subjected to buoyant density sedimentation separation on a single-step Percoll gradient. The recovery of viable cells after Percoll purification was very variable (5–68% of the original total viable cell number) but the purity of the recovered cells was usually high (Table). In 2 cases shown in the Table (T.W. and J.H.) the BAM content was reduced. T.W. was a patient whose bronchoalveolar lavage contained many small, sticky mucus and/or surfactant particles and J.H. suffered from extrinsic allergic alveolitis, the lavage containing many lymphoblasts. Where the PMNL content of the purified BAM population was greater than 5%, such as in the case of T.W., results were not included in the cytotoxicity calculations when obtained from assay wells containing greater than $5 \times 10^3$ PMNL.

*Comparison of cytotoxicity of BAM from patients with and without bronchial carcinoma*

Cytotoxicity assays were set up to test the BAM from some of the patients at a range of E:T ratios from 0·5:1 to 20:1, or as many of these ratios as the cell yield allowed. The cytotoxicity of BAM from the lungs of patients with confirmed chronic bronchitis, cryptogenic fibrosing alveolitis, sarcoidosis and primary bronchial carcinoma is shown in Fig. 2. Data are also included on the cytotoxicity of BAM from patients found on bronchoscopy to be suffering only from mild localized bronchial inflammation. The cytotoxicity of each patient's BAM increased as the E:T ratio was increased to 20:1. When the BAM cytotoxicity values from the non-carcinoma patients were combined at each E:T ratio and compared with those of the bronchial carcinoma-bearing patients, no significant difference between the 2 groups was found at any E:T ratio. Furthermore, in the non-carcinoma group the range of cytotoxicity values at each E:T ratio was composed of an even distribution of data points obtained using cells from all pathologies and there was no consistent high or low trend attributable to any particular disease. In both groups the BAM from individual patients expressed consistently high, medium or low levels of cytotoxicity at each E:T ratio as compared with that of the group as a whole. In one case, there was positive target cell growth enhance-

FIG. 2.—The cytotoxicity of bronchoalveolar macrophages (BAM) from bronchial or non-carcinoma-bearing patients for A549 target cells. The data points on the left-hand side of each effector: target cell ratio column are those obtained from individuals diagnosed as having chronic bronchitis (○), cryptogenic fibrosing alveolitis (●), allergic alveolitis (■), sarcoidosis (△) or mild bronchial inflammation (□). On the right-hand side are those from individuals diagnosed with primary bronchial carcinoma (▽) or secondary melanoma deposits in the bronchus (▼). Each individual's macrophages were tested for cytotoxicity *vs* A549 cells at each effector: target cell ratio. The bars represent means.

ment (negative cytotoxicity) at 0·5:1 and 1:1.

*Is there a biased selection of BAM during purification on Percoll gradients?*

Preliminary experiments indicated that the size and density of BAM were very variable and that the more dense BAM sedimented with the PMNL to a pellet at the bottom of the gradient—being then lost. Many BAM from smokers contain tar-like inclusions in lysosomal bodies (unpublished observations; Hunninghake *et al.*,

FIG. 3.—Cytotoxicity of bronchoalveolar macrophages taken from the Percoll gradient interface (–○–) of pellet (–●–), mean ± 1 s.d. of the mean.

1979; Hocking & Golde, 1979*a*) and, under the conditions of osmolality existing in the Percoll gradients, these cells had a density range comparable to the granular PMNL and sedimented with them. Furthermore, it has been reported by other workers using freshly isolated rodent peritoneal macrophages (Lee & Berry, 1977) or cells adapted to tissue culture (Serio *et al.*, 1979) that there are subpopulations, separable on the basis of their density, whose efficacy as cytotoxic effector cells varies. The observation that the carcinoma group contained more smokers (10/15 compared with 12/24 in the non-carcinoma group) made it important to determine whether the cytotoxicity observed in our experiments was due to a more cytotoxic, less dense subpopulation which was selected on the Percoll gradient as these would constitute a much smaller subpopulation of the BAM of the smoking, bronchial carcinoma-bearing patients. We were fortunate to obtain several lavage fluids that were almost PMNL free (< 3%) and were able, therefore, to compare the cytotoxicity of the BAM isolated from both the Percoll gradient interface and pellet (Fig. 3). The cytotoxicity of both BAM populations increased as the E:T ratio was increased although the mean cytotoxicity of the pellet BAM was higher than that of the interface BAM at the lower E:T ratios and lower at the high E:T ratios. However, at no ratio was this difference statistically significant.

*Can BAM enhance tumour cell growth?*

The potentiation of the tumour target cell growth seen at 0·5:1 and 1:1 with one BAM preparation from a bronchial carcinoma-bearing patient (Fig. 2) raised the question as to whether every BAM population, under certain conditions, might be able to enhance tumour cell growth. The % cytotoxicity of this BAM preparation was consistently very low at each E:T ratio. As the dose–response curves of the more cytotoxic BAM preparations were approximately parallel to this one it was possible that, under conditions where the more cytotoxic BAM preparations were further diluted, enhancement of tumour cell growth might occur. However, only one greatly diluted preparation of BAM (from a sarcoidosis patient) potentiated target cell growth (Fig. 4). The particular BAM preparations from bronchial-carcinoma patients that were used in this experiment appeared unable to do this, suggesting that generally BAM cannot enhance tumour-cell growth *in vitro*.

FIG. 4.—Cytotoxicity of bronchoalveolar macrophages at low cell densities, for A549 target cells. The data points on the left-hand side of each effector:target cell ratio column are those obtained from individuals diagnosed as having allergic alveolitis (■), cryptogenic fibrosing alveolitis (●), mild bronchial inflammation (□) or sarcoidois (△). On the right-hand side are those from individuals diagnosed with primary bronchial carcinoma (▽).

DISCUSSION

The aim of this study was to examine a potential role for human BAM in the host response to bronchial neoplasia. Although the BAM constitutes the most numerous cell type on the bronchial mucosal surface, other potentially cytotoxic effector cells are also present. Many of the patients whose lungs were lavaged had pulmonary abnormalities that were associated with an increased number of lymphocytes and PMNL infiltrating the bronchial lumen from the blood (Hunninghake *et al.*, 1979). On examining whether these "contaminating" cells might affect the results of the BAM cytotoxicity assay it was found that PMNL were cytotoxic for the A549 target cells but lymphocytes were not. The pathological changes accompanying many of the pulmonary diseases and also smoking habits cause variations in mucus and/or surfactant secretion (Finley & Ladman, 1972; Wanner, 1977). These substances affected attempts to purify BAM by adherence to plastic as removal of non-adherent cells was usually accompanied by loss of most of the BAM which adhere avidly in aggregates to the mucus or surfactant (Hocking & Golde, 1979*b*). Purification of human peripheral blood monocytes from lymphocytes and PMNL has been achieved using buoyant density sedimentation in Percoll gradients (Hardin & Down, 1981; Pertoft *et al.*, 1980; Ulmer & Flad, 1979). Percoll was therefore used in an attempt to purify BAM and we found that it was possible to obtain highly purified BAM. BAM are, however, very heterogeneous in their size and density distribution (Hunninghake *et al.*, 1979; Hocking & Golde, 1979*a*; Territo & Golde, 1979; Harris *et al.*, 1970) and in each BAM preparation the density of varying numbers of BAM coincided with that of the PMNL and these cells were lost in the pellet. Studies of rodent peritoneal macrophages have suggested that cytotoxic functions are the property of discrete macrophage subpopulations separable by their velocity of sedimentation in density gradients. In this study the cytotoxicity of the more dense (Percoll pellet) BAM had some characteristics similar to those of peripheral-blood monocytes (Swinburne & Cole, 1982) in that they were more cytotoxic than the less dense (Percoll interface) BAM at low E:T ratios and less cytotoxic at high E:T ratios, although these differences were not statistically significant. This would suggest that the Percoll pellet BAM probably contained a higher proportion of less differentiated cells that had recently entered the bronchial lumen from the blood. These cells are more dense mainly on account of their greater nuclear: cytoplasmic volume ratio. The fact that a more definitive separation was not obtained was probably due to a combination of 3 factors. The first was that the buoyant density separation in this case was partly on the basis of the size of mucus/surfactant-cell aggregates, both high- and low-density cells aggregating to small mucus or surfactant fragments. Second, individual BAM containing tar-like inclusion (presumably more mature cells) are relatively dense and generally sedimented in "the pellet" and, third, there is some *in vitro* differentiation of these less differentiated/more dense cells during the 64h assay period.

Patients undergoing fibreoptic bronchoscopy were routinely anaesthetized locally between the pharyngeal and upper bronchial regions with lignocaine. This has been shown to affect macrophage antibody-dependent cytotoxicity (Kurisu *et al.*, 1978) but whether or not this affects spontaneous cytotoxicity is unknown. However, the fact that variable amounts were administered to each patient, that this was variable and probably greatly diluted as the bronchus divided and its mucosal surfaces were lavaged, and that the BAM isolated from all the different patients were cytotoxic in a similarly dose-dependent manner with the majority exceeding 70% cytotoxicity at 20:1, cumulatively suggests that the cytotoxicity detected here was not affected by pre-exposure of the different preparations of BAM to lignocaine.

Similarly, the fact that the patients studied were very heterogeneous in terms of age, smoking habits, past and present clinical history and drug treatment appears not to have affected the ability of their BAM to be cytotoxic in this assay. However, perturbation of the cytotoxicity of individual BAM preparations, *e.g.* due to patient pretreatment with steroids, cannot be discounted. Of particular interest was the fact that there was no significant difference between the BAM from patients diagnosed as having bronchial carcinoma (the majority of smokers) and other pulmonary disorders (the majority of non-smokers).

BAM were also cytotoxic for two additional human lung cell lines, E14 originating from a human lung squamous-cell carcinoma (Fischer & Wetterlein, 1977) and HS853 (American Type Culture Collection), a culture of fibroblast morphology originating from non-carcinomatous human lung tissue (data not shown). However, whether fresh lung tumour cells, unadapted to tissue culture, are sensitive to BAM was not investigated. However, in this context Vose (1978) has shown that macrophages isolated from within human lung tumours can express cytolytic activity against fresh tumour targets. Furthermore Rhodes *et al.* (1981) have shown that BAM isolated from the vicinity of a malignant bronchial lesion have a reduced Fc-receptor expression and this is possibly due to tumour-derived factors exuded into the bronchial lumen. Whether this may affect the BAM migration towards, infiltration of, and subsequent cytotoxic activity within a bronchial tumour needs further examination. The cytotoxicity of BAM for A549 target cells is known to be due, at least in part, to cytolytic activity (Swinburne & Cole, 1982). Our data are consistent with those of Lemarbre *et al.* (1980) but are not comparable with those of Bordignon *et al.* (1980), who suggested that there is an intrinsic tumoricidal defect in BAM. These discrepancies may be due either to differences in the BAM purification procedures used, *i.e.* selection by adherence or density sedimentation, or to the different target cells used to assess BAM cytotoxicity. Bordignon *et al.* (1980) used SV40 transformed murine kidney-tumour cells as target cells.

Within the non-carcinoma group there were individuals who were bronchoscoped to investigate "non-resolving cough" and haemoptysis but who on bronchoscopy were found to have no more abnormality than a localized mild inflammation of the bronchial mucosa with no obvious cause. Lung lavage in these patients, as with most others, was carried out in an unaffected lobe, and the cytotoxicity of these BAM preparations was similar, both qualitatively and quantitatively, to that of BAM from the various proven pathological states. This would argue against the potent cytotoxicity of BAM detected in this assay being due to a generalized activation relating to smoking habits or a pathological disorder occurring in the bronchi (Hunninghake *et al.*, 1979) but is more probably attributable to an endogenously high state of activation normally present in the BAM.

It has been shown in several studies that under certain conditions macrophages may actually enhance growth of some cell types (Wing & Remington, 1980; Keller, 1975; Leibovich & Ross, 1976; DeLustro *et al.*, 1980: Greenburg & Hunt, 1978) including tumour cells (Binderup *et al.*, 1979; Zipori & Bol, 1979; Nathan & Terry, 1975; Evans, 1979; Mantovani, 1978). It was therefore of interest when one BAM preparation showed significant target-cell growth enhancement. The inability to find further evidence of this in other BAM preparations simply by manipulating the E:T ratio was either indicative of the fact that generally these macrophages were incapable of promoting growth enhancement or that other, as yet undefined, culture conditions are required before this is manifested *in vitro*.

This work was supported by the Medical Research Council and the Board of Governors of Brompton Hospital.

We wish to thank colleagues at Brompton Hospital for their cooperation in obtaining bronchial lavage specimens, Sister Lindsay MacWilliam for clinical coordination and Miss Mary Ash for secretarial assistance.

### REFERENCES

Binderup, L., Bramm, E. & Arrigoni-Martelli, E. (1979) Peritoneal macrophages from adjuvant arthritic rats enhance tumour cell growth *in vitro*. *Experienta*, **35**, 1230.

Bordignon, C., Avallone, R., Peri, G., Poten-Tarutti, N., Mangioni, C. & Mantovani, A. (1980) Cytotoxicity on tumour cells of human mononuclear phagocytes: Defective tumouricidal capacity of alveolar macrophages. *Clin. Exp. Immunol.*, **41**, 336.

Boyüm, A. (1968) Isolation of mononuclear cells and granulocytes from human blood. *Scand. J. Clin. Invest.* **97** (Suppl.), 77.

Brooks, C. F., Rees, R. C. & Robins, R. A. (1978) Studies on the microcytotoxicity test. II. The uptake of amino acids ($^{3}$H-leucine or $^{75}$Se-methionine) but not nucleosides ($^{3}$H-thymidine or $^{125}$I-IUdR) or $^{51}CrO_4^{2-}$ provides a direct and quantitative measure of target cell survival in the presence of lymphoid cells. *J. Immunol. Methods*, **21**, 111.

Cole, P., Turton, C., Lanyon, H. & Collins, J. (1980) Bronchoalveolar lavage for the preparation of free lung cells: Technique and complications. *Br. J. Dis. Chest*, **74**, 273.

DeLustro, F., Sherer, G. K. & LeRoy, E. C. (1980) Human monocyte stimulation of fibroblast growth by a soluble mediator(s). *J. Reticuloend. Soc.*, **28**, 519.

EVANS, R. (1979) Host cells in transplanted murine tumors and their possible relevance to tumor growth. *J. Reticuloend. Soc.*, **26**, 427.

FINLEY, T. N. & LADMAN, A. J. (1972) Low yield of pulmonary surfactant in cigarette smokers. *N. Engl. J. Med.*, **286**, 223.

FISCHER, P. & WETTERLEIN, M. (1977) Establishment and cytogenetic analysis of a cell line derived from a human epithelioma of the lung. *Oncology*, **34**, 205.

GIARD, D. J., AARONSON, S. A., TODARO, G. J. & 4 others (1973) *In vitro* cultivation of human tumors: Establishment of cell lines derived from a series of solid tumors. *J. Natl Cancer Inst.*, **51**, 1417.

GORMAN, N. T. (1979) Alveolar macrophage cytotoxicity in dogs following intravenous BCG. *Eur. J. Cancer*, **15**, 1051.

GREENBURG, G. B. & HUNT, T. K. (1978) The proliferative response *in vitro* of vascular endothelial and smooth muscle cells exposed to wound fluids and macrophages. *J. Cell. Physiol.*, **97**, 353.

HARDIN, J. A. & DOWN, J. T. (1981) Isolation of human monocytes on re-orienting gradients of Percoll. *J. Immunol. Method*, **40**, 1.

HARRIS, J. O., SWENSON, E. W. & JOHNSON, J. E., III. (1970) Human alveolar macrophages: Comparison of phagocytic ability, glucose utilisation and ultrastructure in smokers and non-smokers. *J. Clin. Invest.*, **49**, 2086.

HOCKING, W. G. & GOLDE, D. W. (1979*a*) The pulmonary-alveolar macrophage. *N. Engl. J. Med.*, **301**, 580.

HOCKING, W. G. & GOLDE, D. W. (1979*b*) The pulmonary alveolar macrophage. *N. Engl. J. Med.*, **301**, 639.

HOGG, N. & BALKWILL, F. R. (1981) Species restriction in cytostatic activity of human and murine monocytes and macrophages. *Immunology*, **43**, 197.

HUNNINGHAKE, G. W., GADEK, J. E., KAWANAMI, O., FERRANS, V. J. & CRYSTAL, R. G. (1979) Inflammatory and immune processes in the human lung in health and disease: Evaluation by bronchoalveolar lavage. *Am. J. Pathol.*, **97**, 149.

JERRELLS, T. R., DEAN, J. H., RICHARDSON, G., CANNON, G. B. & HERBERMAN, R. B. (1979) Increased monocyte-mediated cytostasis of lymphoid cell lines in breast and lung cancer patients. *Int. J. Cancer*, **23**, 768.

KELLER, R. (1975) Major changes in lymphocyte proliferation evoked by activated macrophages. *Cell. Immunol.*, **17**, 542.

KURISO, M., YAMAZAKI, M. & MIZUNO, D. (1978) *In vitro* induction of antibody-dependent cytotoxic macrophages by the local anaesthetic lidocaine. *Microbiol. Immunol.*, **22**, 631.

LEE, K. & BERRY, D. (1977) Functional heterogeneity in macrophages activated by *Corynebacterium parvum*: Characterisation of subpopulations with different activities in promoting immune responses and suppressing tumor cell growth. *J. Immunol.*, **118**, 1530.

LEMARBRE, P., HOIDAL, J., VESELLA, R. & RHINEHART, J. (1980) Human pulmonary macrophage tumour cell cytotoxicity. *Blood*, **55**, 612.

LIEBOVICH, S. J. & ROSS, R. (1976) A macrophage-dependent factor that stimulates the proliferation of fibroblasts *in vitro*. *Am. J. Pathol.*, **84**, 501.

LOHMANN-MATHES, M., KOLB, B. & MEERPOHL, H. (1978) Susceptibility of malignant and normal target cells to the cytotoxic action of bone-marrow macrophages activated *in vitro* with the macrophage cytotoxic factor (MCF). *Cell. Immunol.*, **41**, 231.

MANTOVANI, A. (1978) Effects on *in vitro* tumor growth of murine macrophages isolated from sarcoma lines differing in immunogenicity and metastasizing capacity. *Int. J. Cancer*, **22**, 741.

MANTOVANI, A., JERRELLS, T. R., DEAN, J. H. & HERBERMAN, R. B. (1979) Cytolytic and cytostatic activity on tumor cells of circulating human monocytes. *Int. J. Cancer*, **23**, 18.

MOORE, K. & MOORE, M. (1979) Systemic and *in-situ* natural killer activity in tumour-bearing rats. *Br. J. Cancer*, **39**, 636.

NATHAN, C. F. & TERRY, W. D. (1975) Differential stimulation of murine lymphoma growth *in vitro* by normal and BCG-activated macrophages. *J. Exp. Med.*, **142**, 887.

PERTOFT, H., JOHNSONS, A., WÄRMEGÅRD, B. & SELJELID, R. (1980) Separation of human monocytes on density gradients of Percoll. *J. Immunol. Methods*, **33**, 221.

RHODES, J., PLOWMAN, P., BISHOP, M. & LIPSCOMB, D. (1981) Human macrophage function in cancer: systemic and local changes detected by an assay for Fc receptor expression. *J. Natl Cancer Inst.*, **66**, 423.

RODER, J. C., LOHMANN-MATTES, M., DOMZIG, W., KIESSLING, R. & HALLER, O. (1979) A functional comparison of tumour cell killing by activated macrophages and natural killer cells. *Eur. J. Immunol.*, **9**, 283.

RYNING, F. W., KRAHENBUHL, J. L. & REMINGTON, J. S. (1981) Comparison of cytotoxic and microbicidal function of bronchoalveolar and peritoneal macrophages. *Immunology*, **42**, 513.

SERIO, C., GANDOUR, D. M. & WALKER, W. S. (1979) Macrophage functional heterogeneity: Evidence for different antibody-dependent effector cell activities and expression of Fc-receptors among macrophage subpopulations. *J. Reticuloend. Soc.*, **25**, 197.

SWINBURNE, S. & COLE, P. J. (1982) Human peripheral blood monocyte and bronchoalveolar macrophage cytotoxicity for cultured human lung tumour cells. *J. Reticuloendoth. Soc.* (In press).

TERRITO, M. C. & GOLDE, D. W. (1979) The function of human alveolar macrophages. *J. Reticuloend. Soc.*, **25**, 111.

ULMER, A. J. & FLAD, H. D. (1979) Discontinuous density gradient separation of human mononuclear leucocytes using Percoll as gradient medium. *J. Immunol. Methods*, **30**, 1.

VOSE, B. M. (1978) Cytotoxicity of adherent cells associated with some human tumours and lung tissues. *Cancer Immunol. Immunother.*, **5**, 173.

WANNER, A. (1977) Clinical aspects of mucociliary transport. *Am. Rev. Resp. Dis.*, **116**, 73.

WARR, G. A. & RUSSELL MARTIN, R. (1974) Chemotactic responsiveness of human alveolar macrophages: Effects of cigarette smoking. *Infect. Immun.*, **9**, 769.

WING, E. J. & REMINGTON, J. S. (1980) Activated macrophages pre-incubated *in vitro* enhance rather than suppress mitogen-stimulated lymphocyte transformation. *Immunology*, **40**, 239.

YAEGER, H., JR, ZIMMET, S. M. & SCHWARTZ, S. L. (1974) Pinocytosis by human alveolar macrophages: Comparison of smokers and non-smokers. *J. Clin. Invest.*, **54**, 247.

ZIPORI, D. & BOL, S. (1979) The role of fibroblastoid cells and macrophages from mouse bone marrow in the *in vitro* growth promotion of haemopoietic tumour cells. *Exp. Haematol.*, **7**, 13.

ZWILLIG, B. S. & CAMPOLITO, L. B. (1977) Destruction of tumor cells by BCG-activated alveolar macrophages. *J. Immunol.*, **119**, 838.